# Review of case definitions for myalgic encephalomyelitis/chronic fatigue syndrome (ME/CFS)

**DOI:** 10.1186/s12967-020-02455-0

**Published:** 2020-07-29

**Authors:** Eun-Jin Lim, Chang-Gue Son

**Affiliations:** Institute of Bioscience and Integrative Medicine, Department of Korean Medicine, Daejeon University, 62 Daehak-ro, Dong-gu, Daejeon, Republic of Korea

**Keywords:** Myalgic encephalomyelitis, Chronic fatigue syndrome, Systemic exertion intolerance disease, Case definition, Diagnostic criteria

## Abstract

**Background:**

Myalgic encephalomyelitis/chronic fatigue syndrome (ME/CFS) is a debilitating disease with unknown causes. From the perspectives on the etiology and pathophysiology, ME/CFS has been labeled differently, which influenced changes in case definitions and terminologies. This review sought to feature aspects of the history, developments, and differential symptoms in the case definitions.

**Methods:**

A search was conducted through PubMed published to February 2020 using the following search keywords: case definition AND chronic fatigue syndrome [MeSH Terms]. All reference lists of the included studies were checked. Of the included studies, the number of citations and the visibility in the literatures of the definitions were considered for comparisons of the criteria.

**Results:**

Since the first 'ME' case definition was developed in 1986, 25 case definitions/diagnostic criteria were created based on three conceptual factors (etiology, pathophysiology, and exclusionary disorders). These factors can be categorized into four categories (ME, ME/CFS, CFS, and SEID) and broadly characterized according to primary disorder (ME-viral, CFS-unknown, ME/CFS-inflammatory, SEID-multisystemic), compulsory symptoms (ME and ME/CFS-neuroinflammatory, CFS and SEID-fatigue and/or malaise), and required conditions (ME-infective agent, ME/CFS, CFS, SEID-symptoms associated with fatigue, e.g., duration of illness). ME and ME/CFS widely cover all symptom categories, while CFS mainly covers neurologic and neurocognitive symptoms. Fatigue, cognitive impairment, PEM, sleep disorder, and orthostatic intolerance were the overlapping symptoms of the 4 categories, which were included as SEID criteria.

**Conclusions:**

This study comprehensively described the journey of the development of case definitions and compared the symptom criteria. This review provides broader insights and explanations to understand the complexity of ME/CFS for clinicians and researchers.

## Background

Myalgic encephalomyelitis/chronic fatigue syndrome (ME/CFS) is a debilitating disease with core symptoms of fatigue, unrefreshing sleep, postexertional malaise (PEM), and cognitive dysfunction for more than 6 months [1]. This disorder affects individuals of all ages across all socioeconomic, racial, and ethnic groups, ‘approximately estimated 1% of the population, 17 to 24 million people worldwide [2, 3]. The clinical impact of ME/CFS left 27% of the ME/CFS patients bedridden and 29% housebound, leading to 50% unable to work full time and 21% unable to work at all [4]. In 2015, the Institute of Medicine (IOM) in the U.S. announced that ME/CFS is a serious health problem in the form of complex multisystem neurological disorder, which should be the focus of national medical and scientific effort using the recommended name ‘systemic exertion intolerance disorder (SEID)’ [5].

Outbreaks of neurological paralysis-related symptoms with systemic malaise have occurred worldwide (e.g., Los Angeles in 1934 and Iceland 1947, followed by New Zealand and Nevada), and ME/CFS was first acknowledged as a form of 'poliomyelitis' and 'benign ME' in the 1930s and 1950s [6–8]. Later, it was known to be sporadic and not rare in the general population [9]. The etiology of the illness has yet to be revealed, which has led to no established objective diagnostics, pathophysiology or therapeutics [4]. Accordingly, many expert groups have developed case definitions based on clinical features. To date, over 20 ME/CFS case definitions have been established by different groups in various countries [10]. These definitions reflect the historical flow of the clinical features and characteristics of the illness emphasized in a different way according to the perspectives of researchers [11].

Recognition of the changes in the development of the case definitions of ME/CFS is necessary for physicians and researchers to better understand the illness. Differential diagnosis is crucial in the therapeutic process to enhance treatment efficacy; however, a high number of misdiagnoses can lead to delays in the diagnosis and treatment of ME/CFS [12]. Approximately 37% of ME/CFS-like patients had experienced alternative diagnoses, such as psychiatric, pain, or sleep disorders, in clinics [13]. Furthermore, the application of particular case definitions profoundly impacts epidemiological studies of ME/CFS [14]. The prevalence of ME/CFS could widely vary based on the application of case definitions; for example, there were fivefold differences in prevalence using the Fukuda (0.89%) and the Holmes definition (0.17%) [15].

Numerous studies have also documented skepticism among physicians about ME/CFS being a distinct clinical entity, and they do not feel confident in making the diagnosis [12, 16]. One of the reasons is a lack of understanding of ME/CFS, which is likely to result from the complicated backgrounds of this disorder, including indefinite terminologies and etiology. In fact, ME/CFS has been named differently (e.g., postviral fatigue syndrome, neurasthenia) depending on the perspectives of the researchers; likewise, diagnostic criteria or case definitions have also been changed accordingly.

Therefore, this review aims to overview the development of ME/CFS case definitions, which will provide physicians and researchers with a comprehensive picture of the current and prominent features of ME/CFS.

## Methods

### Literature search strategies and data collection

To comprehensively collect the case definitions of ME/CFS, we performed a search through PubMed published to February 2020 and checked all reference lists of the included studies. The following search keywords were used: case definition AND chronic fatigue syndrome [MeSH Terms]. We included studies only for adult populations (> 18 years), and language was limited to English (Additional file 1. Figure S1).

Two authors independently read all the titles, abstracts, and full text retrieved by the search. The literatures were viewed forward the background of case definitions and analyzed according to time-line based changes. Of those included studies, comparisons of the criteria are limited to the eight definitions (Ramsay, International Consensus Criteria (ICC), Holmes, Australian, Oxford, Fukuda, Canadian Consensus Criteria (CCC), systemic exertion intolerance disease (SEID)) that were selected based on the number of Google Scholar citations and the visibility in the literatures (Additional file 1. Table S1).

## Results

### Past and present of ME/CFS

Since the first recognition of ME/CFS in an outbreak in Los Angeles 1934, the illness has undergone various changes in terminology and case definition [17]. Dr. G. Beard (1839–1883) first disclosed the illness in his book and introduced the term 'neurasthenia' in the 1860 s [18]. Later, the features of neurologic symptoms during the U.K. outbreaks led to naming the illness ‘benign ME’ [19], then Ramsay created the 'ME' case definition in 1986 [20]. Serial outbreaks of the illness led to proposing that the condition was linked to viral infection, which altered its name to ‘chronic Epstein-Barr virus syndrome (EBVS)’ in 1982 [21] and 'postviral fatigue syndrome (PVFS)' in 1985 [22]. In 1988, insufficient evidence in connection with the virus and numerous sporadic cases in the general population led to the Centers for Disease Control and Prevention (CDC) to create the new term ‘CFS' (Holmes definition), which was proposed to more inclusively describe the symptom complex, including psychological symptoms [23]. In 2003, the 'ME/CFS' by CCC was published embracing the clinical features of both 'ME' and 'CFS' [24]. The conception of 'ME' or 'ME/CFS' adopted the notion of neuroinflammation [24, 25].

The terminologies of ME, CFS, and/or ME/CFS have been and interchangeably used until present. The complexity of those indefinite terminologies is shown in the international code for disease (ICD) system. The WHO initially classified the illness as a neurological disorder in the ICD-8 (code 796.0) in 1969 [26]. Subsequently, the ICD-10 (2016) classified it as PVFS (code G93.3) indexing only ‘benign ME’ [27]—‘benign’ was dropped in the 2019 version [28]. Herein, ‘CFS’ was not coded; therefore, clinicians instead often used the code of malaise and fatigue (R53), fatigue syndrome (F48) or even neurasthenia (F48) [28, 29]. Meanwhile, the latest version ICD-11 (2019) includes both ‘(benign) ME’ and ‘CFS’ under PVFS (code 8E49), which noticeably specified exclusion of fatigue (MG22) from the category [30]. This implies that ME/CFS is still loosely defined, yet the perspective of ‘CFS’ has diverged from ‘fatigue’, and there was an attempt to view the illness (ME and CFS) as one disorder by using the same ICD code.

In addition, the mischaracterization of the illness in relation to psychological or primarily fatigue-related disorders has contributed to confusion in using the terms. Contrary to the initial 'neurasthenia' that indicated an organic neurological disease, the term was coopted as 'neurosis' that was indicative of psychiatric origin by Dr. S. Freud (1856–1939), who believed the illness was caused by unresolved conflicts in the unconscious mind [31]. Similarly, in the 1970s, McEvedy alleged its psychological origin with the term ‘myalgia nervosa’ [32]. The debate on the origin of illness (psychological *versus* neurological) seemed to be controversial until recently [8]. Approximately 20% of the U.S. media articles during 1987–2013 mislabeled ME/CFS as 'fatigue or psychosomatic-related disorder', which trivialized the illness [33]. Currently, the disorder is generally considered a complex, multisystem neuroimmune disease [34]. In 2015, the IOM suggested a new term SEID and its criteria, thereby reducing the perception derived by the word 'fatigue' and focusing more on the core symptoms that systemically manifest as a physical illness [5].

### Outline of the development of the case definitions

**Fig. 1:**
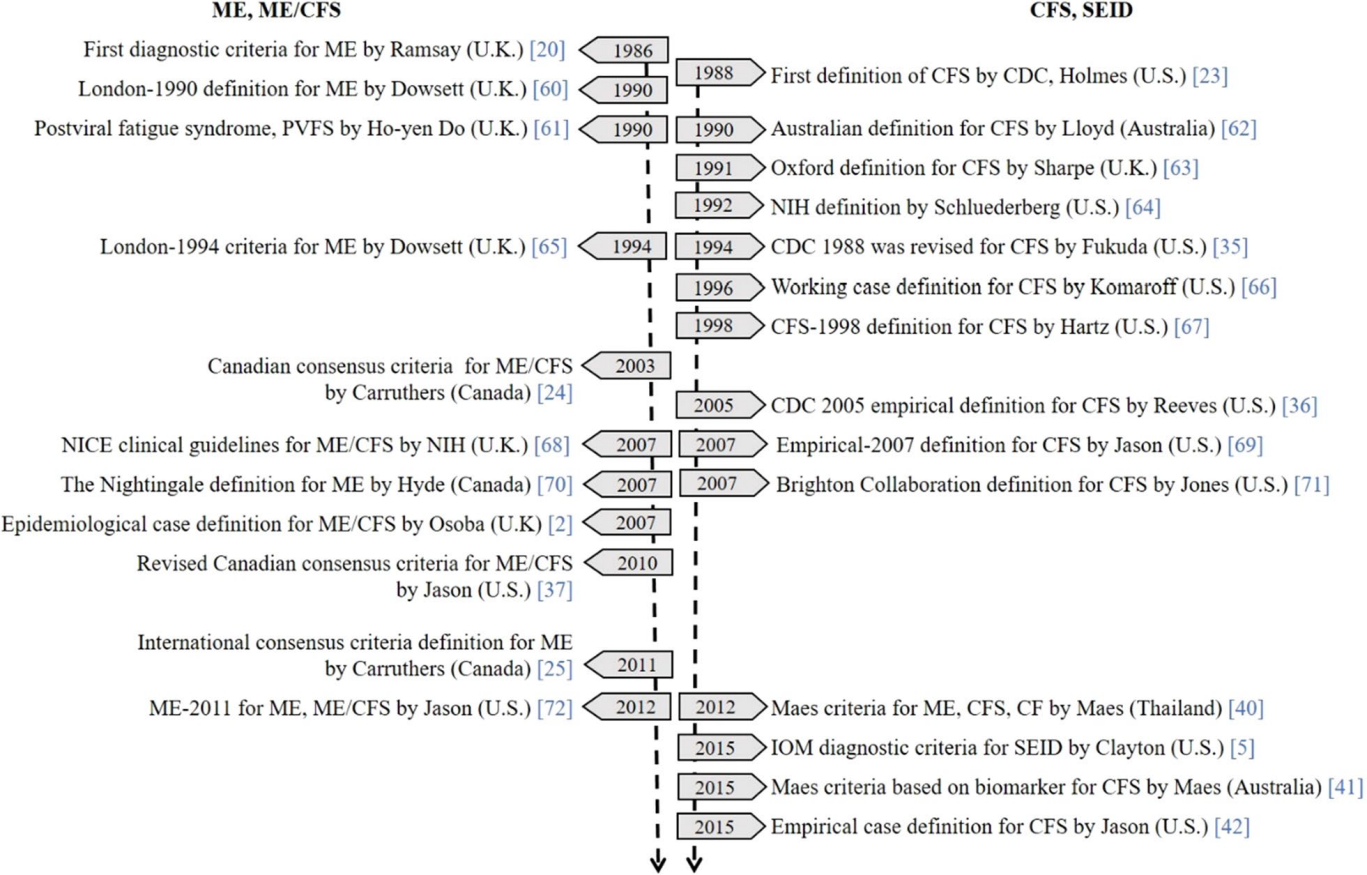
Developmental timeline of ME/CFS case definitions and terminologies. *ME/CFS* myalgic encephalomyelitis and chronic fatigue syndrome, *CDC* Centers for Disease Control and Prevention, *NICE* National Institute for Health and Clinical Excellence, *IOM* Institute of Medicine, *SEID* systemic exertion intolerance disease. Case definition, a specific set of criteria used to define a disease for surveillance. Diagnostic criteria, guidance to indicate the presence of an illness (signs and symptoms, test results)

As shown in Fig. 1, to date, 25 case definitions have been developed and published in English. As regards the unique historical backgrounds in the development of these case definitions, we allocated the definitions into four categories based on characteristics and three by time period.

First, they were divided into ME (mostly published in the U.K.), ME/CFS (mostly Canada), CFS (U.S. and Australia) and SEID (U.S.). The initially distinct view of ME and CFS was later reformulated into ME/CFS and SEID. The feature of these categories is linked to the suggested etiologies: ME by a viral infection, ME/CFS by a neuro-inflammatory disorder, CFS with unknown cause, and SEID by multisystemic effects [5, 20, 24, 35]. The compulsory symptoms in the ME and ME/CFS criteria stressed neuroinflammation, whereas the symptoms for CFS and SEID focused more on fatigue or malaise [5, 20, 24, 35]. While the ME criteria required the presence of an infective agent, the CFS criteria required the conditions of symptoms associated with fatigue (e.g., duration of the illness).

Second, the definitions can be divided into three groups by the developmental approaches, chronologically: 1986–1998, 2003–2011, and 2012–2015. By 1998, the ME and CFS definitions were distinctly created and revised based on clinical case reports or committee consensus. In 2003, the merged form of ME/CFS was developed, which first adopted the empirical synthetic strategy using the experiences of physicians and experts [24]. From 2003 to 2011, it was the most evolving period of its development, shifting to empirically derived definitions. For example, CDC-2005 suggested the use of 3 standardized tools, the fatigue-scoring scales; the Medical Outcomes Survey Short Form-36 (SF-36), the Multidimensional Fatigue Inventory (MFI), and the Symptom Inventory (SI) to evaluate symptoms [36]. Additionally, Osoba generated an epidemiological case definition (ECD) based on patient data provided by physicians in 2007 [2]. Similarly, a questionnaire assessing the severity and frequency of the symptoms was adapted to operationalize the key symptoms in development of the revised CCC definition in 2010 [37]. Of interest, in 2011, the CCC extensively changed the definition for ICC-ME, with a greater focus on inflammatory and neurological symptoms [25], however, the lack of evidence of inflammation was problematic [38, 39]. Since 2012, novel strategies using empirical approaches have been recommended, for example, applying statistical analyses of the symptom patterns or comparisons of biomarkers across subgroups of ME/CFS patients [40–42].

### Comparison of the symptoms and scope of case definitions

**Fig. 2:**
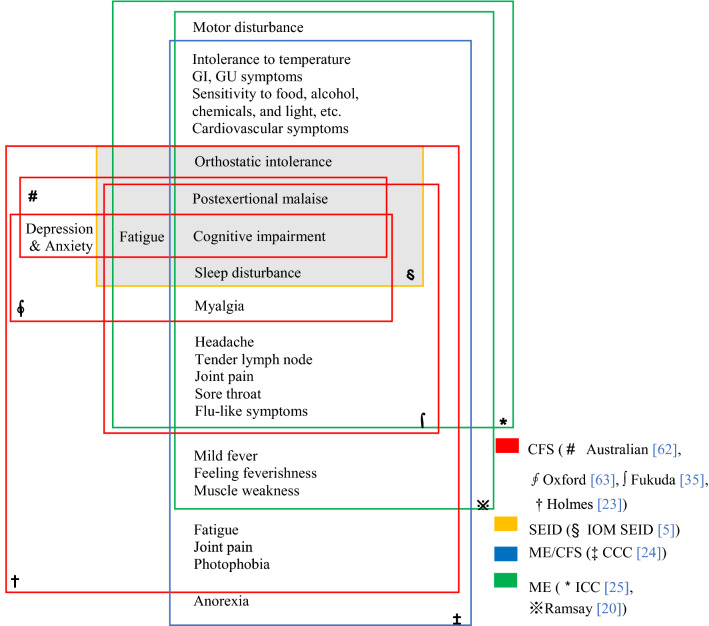
Scope of ME/CFS symptoms by case definitions. CFS, chronic fatigue syndrome, IOM, Institute of Medicine. SEID, systemic exertion intolerance disorder. *ME* myalgic encephalitis, *CCC* Canadian Consensus Criteria, *ICC* International Consensus Criteria, *GI* gastro-intestinal, *GU* genito-urinary symptoms

As shown in Fig. 2, we selected the eight most prominently cited case definitions and diagnostic criteria (in descending order of the citation: Fukuda, Holmes, Oxford, CCC, ICC, Australian, Ramsay, and SEID) from the 25 case definitions. These definitions can also be categorized into ME (ICC and Ramsay), ME/CFS (CCC), CFS (Holmes, Australian, Oxford, Fukuda), and SEID, according to the focus of primary disorder. ‘Cognitive impairment’ is the core symptom that commonly intersected in the eight case definitions. In regard to the ME case definitions, the ICC focused on ‘physical and cognitive fatigability’, while Ramsay particularly emphasized ‘muscle fatigability’. Among the CFS definitions, the Australian definition contains the loosest criteria (fatigue, cognitive impairment), in contrast to the Holmes definition that restrictively embraces the other CFS criteria of the Australian, Oxford, Fukuda, and SEID. Those CFS definitions include depression and anxiety in the criteria, unless presented as primary disorder for Fukuda and SEID. The CCC criteria involved both symptom characteristics of ME and CFS, including ‘anorexia’. These differences in criteria impact the prevalence rates even in the same population; for example, the rates were 0.19% with Fukuda, 0.11% with CCC, and 0.03% with ECD, among 143,153 participants in the U.K.[14]. Meanwhile, the five symptoms ‘fatigue, cognitive impairment, PEM, sleep disorder, and orthostatic intolerance’ overlapped with the 4 categories of ME, ME/CFS and CFS, and SEID. In fact, these symptoms are the core signs of SEID [5]. In general, CFS and SEID definitions focused on ‘cognitive impairment and fatigue’, whereas the ME and ME/CFS further emphasized muscle disturbance with neuro-autonomic symptoms such as sensitivity to food, chemicals or light.

Each case definition generally comprised three categories: required conditions, inclusions and exclusionary symptoms/disorders. Illness ≥ 6 months, unexplained fatigue, and ≥ 50% reduced daily activity are commonly required for the CFS, ME/CFS, and SEID definitions, while the ME definitions require the presence of an infective agent (no requirement of 6 months of illness). The above eight definitions have 30 symptoms in the inclusion criteria that can be subcategorized into five groups: 9 neurologic, 6 neurocognitive, 2 neuroendocrine, 5 autonomic, and 7 immunologic symptoms. The ICC, CCC, Holmes, and Fukuda need 4 to 8 symptoms to meet the criteria (Table 1). The ME (Ramsay, ICC) and ME/CFS (CCC) involve all five subcategories, while CFS definitions (Holmes, Australian, Oxford, and Fukuda) cover mainly the neurologic and neurocognitive symptoms. Regarding the exclusionary criteria, CFS, ME/CFS, and SEID definitions recognized depression and anxiety as possible symptoms or comorbidities of the illness, while the ME (Ramsay, ICC) criteria considered those symptoms as exclusions (Table 1).

**Table 1:** Comparisons of ME/CFS criteria and symptoms by case definitions

Criteria	ME		CFS				ME/CFS	SEID
	Ramsay	ICC	Holmes	Australian	Oxford	Fukuda	CCC	IOM
Required conditions								
Duration (months)			** ≥ **6	** ≥ **6	** ≥ **6	** ≥ **6	** ≥ **6	** ≥ **6
New onset (not lifelong)	Infectious		⊙		⊙	⊙	⊙	⊙
Fatigue (unexplained)			●	●	●	●	⊙	⊙
Reduced daily activity (%)		** ≥ **50	** ≥ **50	●	** ≥ **50	●	⊙	⊙
Infective agent	●	●			●			
* N.* of symptoms required		** ≥ **8 ** ≥ **1 (C/D), ** ≥ **1 (D/E), ≥ **1 (**B/C)	** ≥ **8(1** ≥ **B)			** ≥ **4	** ≥ **7 ** ≥ **2 (A/B), ≥ **1 (**C/D/E)	** ≥ 1 (**B/D)
No result of physical exertion						●		●
No result of mental exertion						●		●
No alleviation by rest			●			●		●
Postexertional malaise (≥ 24 h)	○	●	⊙	○		⊙	●	●
Inclusions								
A. Neurologic								
Myalgia	○		⊙		○	⊙	⊙	
Muscle weakness	●	⊙	⊙					
Motor disturbance	○	⊙						
Generalized hyperalgesia		⊙						
Joint pain		⊙	⊙			⊙	○	
New/headaches	○	⊙	⊙			⊙	○	
Disturbed sleep patterns	○	⊙	⊙		○		⊙	
Unrefreshing sleep		⊙			○	⊙	○	●
Drowsiness		⊙			○		○	
B. Neurocognitive								
Difficulty thinking/processing		⊙	⊙	○			⊙	
Short-term memory loss	○	⊙	⊙	●	○	⊙	⊙	⊙
Difficult to focus	○	⊙	⊙	○		⊙	⊙	
Depression/anxiety			⊙	○	○			
Hypersensitivity to noise/light	○	⊙	⊙				⊙	
Tinnitus, double vision	○						⊙	
C. Neuroendocrine								
Thermostatic instability	○	⊙					⊙	
Anorexia							⊙	
D. Autonomic dysfunction								
Orthostatic intolerance							⊙	⊙
Cardiovascular	○	⊙					⊙	
Respiratory	○	⊙					⊙	
Gastro-intestinal (GI)	○	⊙					⊙	
Genito-urinary (GU)	○	⊙					⊙	
E. Immune								
Fever or chills	○		⊙					
Flu-like symptoms	○	⊙					⊙	
Susceptibility to virus		⊙						
Sore throat	○		⊙			⊙	⊙	
Lymph node pain/tenderness	○		⊙			⊙	⊙	
Sensitivity to chemicals, foods, medications, odors		⊙					⊙	
Exclusions								
Medical conditions cause chronic fatigue	x		x	x	x	x	x	
Psychiatric disorders	x	x	x	x	x	x	x	
Primary brain disorders			x	x	x	x	x	
Substance abuse, eating disorder		x	x	x	x	x	x	
Active process of disease		x					x	
Reactive depression		x						
Depression and anxiety	x							

*CFS* chronic fatigue syndrome, *ME* myalgic encephalitis, *CCC* Canadian Consensus Criteria, *ICC* International Consensus Criteria, *IOM* Institute of Medicine, *SEID* systemic exertion intolerance disorder

● Compulsory/major symptoms, ⊙ Optional/minor symptoms, ○ Inclusive symptoms, x Excluded symptoms

## Discussion and conclusions

As we described above, the current status of the illness might be linked to the unique historical background of ME/CFS. The decades of effort to unearth this illness is well reflected in the development of case definitions and terminologies. In this review, we found three key factors that have affected ME/CFS case definitions: etiology, pathophysiology, and exclusionary disorders. These factors have impacted the specification of the main symptoms, required conditions, and range of inclusive and exclusive symptoms/disorders in the development of case definitions (Table 2).

**Table 2:** Summary of classification for the ME/CFS case definitions

Items	Categories			
	ME	ME/CFS	CFS	SEID
*N.* of case definitions (Country)	11 (U.K., Canada)		14 (U.S., Australia)	
Author	Ramsay	Carruthers	Holmes	Clayton
Publication year	(1986)	(2003)	(1988)	(2015)
Most cited eight case definitions	Ramsay, ICC	CCC	Holmes, Australian, Oxford, Fukuda	IOM
Primary disorder	Viral	Inflammatory	Unknown	Multisystemic
Compulsory symptom	Neuroinflammatory symptoms (e.g., muscle disturbances)		Fatigue and/or malaise	
Required conditions	Infective agents	Symptoms associated with fatigue (e.g., duration of the illness)		
Depression and anxiety	Excluded	Inclusive		
Coverage of symptoms	All five symptom categories*		Mainly neurologic and neurocognitive symptoms	
Common symptomof the case definitions	Cognitive impairment			
Common symptoms of the categories	Fatigue, cognitive impairment, sleep disorders, orthostatic intolerance			

*Five symptom categories: neurologic, neurocognitive, neuroendocrine, autonomic dysfunction, and immune

Infection, genetics, and environmental factors including trauma, are the most commonly discussed etiologies of ME/CFS, and infection has long been debated as one of the triggers since the initial outbreaks [43]. Some studies have reported the partial linkage between certain viral infections and the development of ME/CFS [44, 45]; however, the association of virus and this illness has not yet been established. Recently, novel hypotheses have proposed virus-induced alterations in mitochondrial metabolism [46] and autoimmune systems [47, 48]. These hypotheses are related to some pathophysiologic features involving impairments in the central and autonomic nervous system (CNS and ANS), metabolic function and immunologic system [49]. Recent clinical data support those hypotheses that found widespread neuroinflammation by microglial activation in PET scans [50], lower levels of metabolites [51], and unique patterns of inflammatory cytokines according to the severity in ME/CFS patients [52]. Additionally, as no single cause has been found, multifactorial contributors (e.g. trauma, toxin exposure, and genetic susceptibility) were suggested [53].

Meanwhile, these various and undefined pathophysiology strongly suggest the possibility of heterogeneous or subsets of ME/CFS [29]. From the empirical analyses of patient symptoms, the neurologic and neurocognitive symptoms were identified as the core symptoms across the major eight definitions [41, 42]. Accordingly, some research groups have tried to classify ME/CFS patients into subgroups for pathophysiologic studies [52, 54, 55]. In fact, a case definition-based diagnosis is problematic, especially for disorders with heterogeneous and unknown underlying pathologies, such as ME/CFS [56]. For the diagnosis of those disorders, the use of diagnostic criteria instead of case definitions are recommended as a more suitable method [56]. Our recent meta-analysis found highly varied prevalence rates of ME/CFS according to the definitions, e.g., rates with Oxford, 1.41%; Fukuda, 0.89%; Australian, 0.79%; and Holmes, 0.17% [15]. This is possibly due to unreliable selection of the homogeneous patient group [10]. The existence of subgroups might lead to continuous changes in the ME/CFS definition, and it may be difficult to differentiate these groups with a diagnosis within a case definition.

Most studies and clinicians adapted those case definitions (instead of diagnostic criteria) since the first case definition in 1988; however, they have been criticized from various aspects. For example, the polythetic method (selection 4 out of 8 symptoms) of the Fukuda definition has been claimed to be problematic due to the possibility of misdiagnosis or overdiagnosis of the illness [42]. The Holmes, ICC, and CCC definitions were not exempt from those claims [10]. Hence, in 2015, the IOM announced ‘SEID diagnostic criteria’ consisting of the diagnostic algorithm based on the core symptoms [5]. Although, SEID has also been criticized for the possibility of increasing prevalence rate [57], SEID criteria seems to be well-matched with the recent findings, such as mild neuro-inflammation and lower levels of metabolites [49–52]. One study found a 2.8-fold increase in the number of ME/CFS cases with the SEID criteria compared to the Fukuda definition [57].

The perspectives of researchers on etiology and pathophysiology of the illness have influenced on the case definitions, which have been continuously changed. It may be unable to avoid the changes, unless the etiology of ME/CFS is revealed. In this study, we classified the definitions into four concepts and probed the developmental changes in timeline basis. Also, of the compared definitions, it was remarkable that the neurologic and neurocognitive symptom were overlapped among the complex definitions. The possibility of the heterogeneous characteristics of the illness may have critical limitations in case definitions, and then urgently requires the development of objective diagnostic tools for ME/CFS. It is promising that a biological measurement tool using a blood sample-derived nanoelectronics assay could differentiate patients with ME/CFS from controls [58]. In line with this, to objectively assess PEM, a key symptom of ME/CFS, a standardized technique to measure the level of oxygen uptake using cardiopulmonary exercise testing (CPET) has recently been developed [59]. In addition to the development of advanced diagnostic tools, fine study design or strategies such as the well-constructed patient database, prospective cohort studies and clinical trials for the objective measurement of the core symptoms in particular are needed to comprehensively understand the illness [1, 29, 49].

In summary, we have comprehensively reviewed the case definitions and the complicated journey in the developments. We herein found the vital differences and similarities among those definitions, particularly the eight definitions that was most likely to be used in research and clinical practice. We didn’t intend to seek for a better case definition among them, instead, attempted to shed light on the complexity and confusion of ME/CFS. This review would provide broader insights to understand this complex illness for clinicians and researchers.

## Supplementary information

**Additional file 1: Table S1** Citations for case definitions of ME/CFS. **Figure S1** Flow chart of the study selection process.

## Data Availability

All data related to this study are available in the public domain.
