# Supplementary material for: Review of case definitions for myalgic encephalomyelitis/chronic fatigue syndrome (ME/CFS)
Source: J Transl Med. 2020 Jul 29;18:289. doi: 10.1186/s12967-020-02455-0 (PMC7391812; doi:10.1186/s12967-020-02455-0)
Supplement: Supplementary file 1 — Additional file 1: Table S1 Citations for case definitions of ME/CFS. Figure S1 Flow chart of the study selection process. [file 12967_2020_2455_MOESM1_ESM.docx]

| **Supplementary Table 1. Citations for case definitions of ME/CFS** | | | | | |
| --- | --- | --- | --- | --- | --- |
| **#** | **Case definitions** | **Author** | **Year** | **Country** | **Citations*** |
| 1 | ME diagnostic criteria [20] | Ramsay | 1986 | U.K. | 146 |
| 2 | CDC-1988 (CFS) [23] | Holmes | 1988 | U.S. | 2326 |
| 3 | London-1990 (ME) [60] | Dowsett | 1990 | U.K. | 176 |
| 4 | Postviral fatigue syndrome (PVFS) [61] | Ho-yen Do | 1990 | U.K. | 37 |
| 5 | Australian definition (CFS) [62] | Lloyd | 1990 | Australia | 500 |
| 6 | Oxford definition (CFS) [63] | Sharpe | 1991 | U.K. | 1121 |
| 7 | NIH definition (CFS) [64] | Schluederberg | 1992 | U.S. | 385 |
| 8 | London-1994 (ME) [65] | Dowsett | 1994 | U.K. | 28 |
| 9 | CDC-1994 (CFS) [35] | Fukuda | 1994 | U.S. | 5548 |
| 10 | Working case definition (CFS) [66] | Komaroff | 1996 | U.S. | 206 |
| 11 | CFS-1998 (CFS) [67] | Hartz | 1998 | U.S. | 28 |
| 12 | Canadian consensus criteria (ME/CFS) [24] | Carruthers | 2003 | Canada | 839 |
| 13 | CDC-2005 empirical definition (CFS) [36] | Reeves | 2005 | U.S. | 309 |
| 14 | NICE clinical guidelines (ME/CFS) [68] | NIH | 2007 | U.K. | 46^†^ |
| 15 | Empirical-2007 (CFS) [69] | Jason | 2007 | U.S. | 32 |
| 16 | Brighton collaboration definition (CFS) [71] | Jones | 2007 | U.S. | 20 |
| 17 | The Nightingale definition (ME) [70] | Hyde | 2007 | Canada | 19 |
| 18 | Epidemiological case definition (ME/CFS) [2] | Osoba | 2007 | U.K. | 9 |
| 19 | Revised Canadian consensus criteria (ME/CFS) [37] | Jason | 2010 | U.S. | 133 |
| 20 | International consensus criteria (ME) [25] | Carruthers | 2011 | Canada | 642 |
| 21 | ME-2011 (ME, ME/CFS) [72] | Jason | 2012 | U.S. | 57 |
| 22 | Maes criteria (ME, CFS, CF) [40] | Maes | 2012 | Thailand | 70 |
| 23 | IOM diagnostic criteria (SEID) [5] | Clayton | 2015 | U.S. | 135 |
| 24 | Maes criteria on biomarker (CFS) [41] | Maes | 2015 | Australia | 7 |
| 25 | Empirical case definition (CFS) [42] | Jason | 2015 | U.S. | 30 |
| *number of Google scholar citations, searched 19 November 2019. ^†^Summary of NICE guidance BMJ 2007;335:466. | | | | | |

PubMed searched

(n = 851)

Excluded (n = 16)

No full text and not related: 7

Duplicated articles: 5

Not in English: 4

Excluded (n = 25)

Biological studies of ME/CFS: 4

Study methodologies: 16

Epidemiology studies: 5

Articles after title search

(n = 91)

Excluded (n = 23)

Comparison of case definitions: 7

Treatment and prevalence: 8

Psychiatric and opinion: 8

Excluded (n = 17)

Survey questionnaire and case reports: 6

Review of case definitions: 11

Articles met inclusion criteria

(n = 25)

Articles after abstract search

(n = 50)

Articles added from reference lists and Google search

(n = 15)

**Supplementary Figure 1. Flow chart of the study selection process**
